# Erratum for Mann et al., “Heterogeneous lineage-specific arginine deiminase expression within dental microbiome species”

**DOI:** 10.1128/spectrum.00347-25

**Published:** 2025-02-27

**Authors:** Allison E. Mann, Brinta Chakraborty, Lauren M. O'Connell, Marcelle M. Nascimento, Robert A. Burne, Vincent P. Richards

## ERRATUM

Volume 12, no. 4, e01445-23, 2024, https://doi.org/10.1128/spectrum.01445-23. Page 22, Acknowledgments: “…grant numbers P20GM146584 and P20GM139767” should read "…and P20GM139769."

