# Supplementary material for: Heterogeneous lineage-specific arginine deiminase expression within dental microbiome species
Source: Microbiol Spectr. 2024 Feb 27;12(4):e01445-23. doi: 10.1128/spectrum.01445-23 (PMC10986539; doi:10.1128/spectrum.01445-23)
Supplement: Figures S1 to S4 — All supplemental figures. [file spectrum.01445-23-s0001.docx]

Heterogeneous lineages-specific arginine deiminase expression within dental microbiome species

Allison E. Mann, Brinta Chakraborty, Lauren M. O’Connell, Marcelle M. Nascimento, Robert A. Burne, Vincent P. Richards

**Supplementary Figures:**

**FIG S1.** Correlation of citrulline production with the abundance of *Streptococcus mutans* as measured by ASV relative abundance. Trend lines are shown for each group (PD or PF), R^2^ value and p-value on graph adjusted for both groups.

**FIG S2.** (a) Volcano plot of ADS genes (collapsed by species) up or down regulated in PD samples with high (>5%) or low (<5%) abundance of *Streptococcus mutans* as measured by *rpo*C sequencing. (b) Corresponding principal coordinate plot illustrating the ADS gene expression similarity between individual PD samples colored by *S. mutans* abundance group.

**FIG S3**. Relative abundance of two ADS competent species, *S. oralis* and *S. sanguinis* in PF and PD samples with high (>5%) or low (<5%) *S. mutans* abundance.

**FIG S4.** Rarefaction analysis of all samples.
